# Yoga and Physical Rehabilitation Medicine: A Research Partnership in Integrative Care

**DOI:** 10.4172/2157-7595.1000149

**Published:** 2013-12-07

**Authors:** KR Middleton, AT Acevedo, L Dietz, Z Brandon, R Andrade, GR Wallen

**Affiliations:** 1 National Institutes of Health (NIH), Clinical Center, 10 Center Drive, Room 2B02E, Bethesda, USA; 2 NIH/ Rehabilitation Medicine Department, 10 Center Drive, Bethesda, USA

**Keywords:** Yoga, Minority, Osteoarthritis, Rheumatoid arthritis, Physical rehabilitation medicine

## Abstract

Mind-body interventions, such as yoga, that teach stress management with physical activity may be well suited for investigation in both osteoarthritis and rheumatoid arthritis. In order to be considered as viable care options integrative studies need to offer a comprehensive design and include clinicians familiar with the disease process of the study populations. A review of the literature reveals a dearth of information related to the collaboration between yoga and physical rehabilitation medicine. This article discusses the collaboration with physical rehabilitation medicine to collect relevant pre- and post-intervention measures for an on-going pilot acceptability/feasibility yoga study for minority patients with osteoarthritis or rheumatoid arthritis.

An interdisciplinary clinical research team selected psychosocial and physical measures for a community sample of bilingual minority patients, not typically identified as practicing yoga. Sixteen female adults aged 40–63 years (mean =51) completed baseline physical assessments using single leg stance, functional reach test, time up and go test, timed up from the floor test and the Disabilities of the Arm, Shoulder and Hand measures. Baseline values show an average level of functional ability prior to beginning the intervention. Preliminary results indicate some improvement; however, selected measures may not have the sensitivity and specificity needed to identify significant change. In this study, combining interdisciplinary perspectives enhanced the quality of the research study design. The experience of this interdisciplinary clinical research team opens the discussion for future collaborations.

## Introduction

There is a need for improvement in the design of research on integrative interventions for arthritis. The purpose of this article is to share the experience of collaboration with physical rehabilitation medicine in designing and implementing a pilot feasibility study of yoga as an integrative approach to the self-care and functional capacity issues associated with arthritis. The pilot study also obtains information regarding the acceptability of yoga for a community sample of urban, bilingual minority patients who typically have not been identified as practicing yoga or participating in yoga research. This study attempts to fill some of the gaps cited within critiques of other published yoga research. Information regarding the methodology, literature review and creation of the pilot study are detailed in our original article [1].

The ongoing study follows minority adults diagnosed with either osteoarthritis (OA) or rheumatoid arthritis (RA) as they undergo an 8-week program of yoga classes twice a week for 60-minute sessions. The yoga series focuses on the branch of Hatha yoga that uses postures (asanas), breathing techniques (pranayama) and meditation [1]. Classes are bilingual (English/Spanish), and taught by racially concordant yoga teachers. Classes are kept to five participants to allow for detailed modifications and use of props, as needed to tailor the yoga poses to the needs and limitations of every individual.

The interdisciplinary approach to this study brings together yoga practitioners and clinicians with expertise in research, nursing, physical rehabilitation medicine, and rheumatology to create and implement a study that includes quantitative measures related to physical ability and psychosocial patient reported outcome measures. Physical measures are assessed at two time points in an attempt to quantify changes related to participation in an 8-week yoga series.

## Background

When reviewing the literature to create the study, it was observed that yoga interventions have been shown to produce improvements in physiological and quality-of-life measures related to sense of wellbeing, energy, and fatigue; as well as beneficially impacting mood, depression and anxiety disorders [1]. Studies were found related to measuring the effect of yoga on specific joints (knees and hands) [2,3]. Global physical measures testing the impact of practicing yoga were not readily available; therefore, the expertise of physical rehabilitation medicine was enlisted.

It is not apparent if this type of collaboration has previously been attempted, a review of the literature revealed a dearth of information. An article was found which explored the application of modified yoga techniques, as an adjunct to voice therapy, by a speech pathologist who is also a yoga teacher [4]. A randomized control pilot study was conducted in the neurological rehabilitation unit of a university research hospital, to study the effects of pranayama and meditation in rehabilitation of patients with Guillain-Barre syndrome [5]. An Australian based article reported on the first study about the attitudes and behavior of Australian rehabilitation physicians to complementary and alternative medicine (CAM). The article reported knowledge about and referrals to CAM therapies and their perceived effectiveness, with yoga among the most commonly prescribed therapies [6].

According to a 2012 article, forty percent of International Association of Yoga Therapists’ members were professionals within specializations in one or more fields of Western medicine [7]. As yoga therapy grows in popularity, an increasing number of practitioners, including physical and occupational therapy are integrating therapeutic yoga into their practice. According to the therapists on our team, there has been an increase in continuing education options for occupational and physical therapists, which combine yoga and physical rehabilitation therapy. There appears to be a trend towards using the integrative modalities, such as yoga, as modifications for use within a therapeutic practice.

## Collaboration and Measures

During the development of this study, it was not clear which specific measures would best evaluate the physical benefits of regular yoga practice. In an attempt to include relevant physical measures pre- and post-intervention, the Rehabilitation Medicine Department (RMD) at the National Institutes of Health (NIH) Clinical Center was consulted to collaborate on the creation and implementation of the study. Their expertise was sought in order to determine the most appropriate measures related to strength, motion, endurance, and balance, as potential outcome measures for this population and the yoga intervention. Previous experience in the clinical setting between rheumatology, nursing, and rehabilitation medicine suggested this would be an ideal collaboration in order to obtain a more empirical and clinical perspective when evaluating changes over time.

It was necessary to talk through concerns expressed by the physical medicine rehabilitation clinicians in terms of the safety and appropriateness of yoga poses for this population. The team attended a presentation and later a demonstration yoga class given by Dr. Haaz, a researcher and yoga therapist, who designed the series of yoga poses. Her previous research tested the efficacy of the series of yoga in a population with arthritis at Johns Hopkins University [8]. As this research study took shape, interdisciplinary members met to exchange ideas and experience of working with the target population. Once clinicians interested in serving as Associate Investigators were identified, their input was solicited to write sections of the study and to determine the patient flow through the rehabilitation assessment phase of the study.

Clinical measures were selected to evaluate domains of balance and functional mobility that may be responsive to change with an exercise intervention (Table 1). For this pilot study, the physiatrist completes a history and physical, which includes manual muscle testing as part of the neurological and musculoskeletal examination (range of motion, strength, and flexibility). This examination gives a clinical picture of the participant’s strength in all major muscle groups and identifies potential concerns about functional ability and balance prior to participating in the study. The physiatrist also completes the single leg stance (SLS) [9], functional reach test [10] and the timed “*Up and Go*” *test* (TUG) measures [11].

Physical therapy uses a timed floor-transfer test to measure strength, flexibility, function, and problem solving [12]. For this study, participants are timed as they transfer from supine on the floor to standing in any way that they are able. The evaluation concludes with occupational therapy completing the Disabilities of the Arm, Shoulder, and Hand (DASH) [13]. Other yoga and arthritis studies have documented using grip strength and other hand measures [2], but the DASH was selected because it is a global measure of upper body function. The same comprehensive assessment is repeated again by rehabilitation medicine after each participant completes the yoga classes. The initial draft of the study included more measures, however upon suggestion from outside reviewers; the number of measures was reduced for this pilot study.

## Study Population

**Figure 1:**
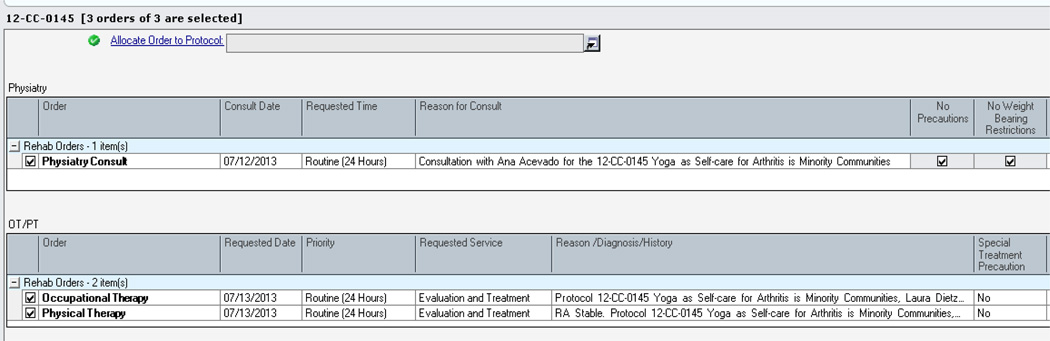
Sample of Referral.

Research participants are recruited from English-speaking or Spanish-speaking patients receiving care from the National Institute of Arthritis and Musculoskeletal and Skin Disease (NIAMS) Community Health Clinic (CHC) located in the Washington, DC area, and those already enrolled on the NIAMS Natural History of Rheumatic Disease in Minority Communities study. Patients already receiving care through NIAMS rheumatology and/or rehabilitation medicine are referred to the yoga study. Once eligibility is verified and consent forms are signed, patients are seen by rehabilitation medicine for a baseline assessment of functional and physical ability. In order to streamline the referral process for the rheumatologists, an order set was created Figure 1 to have the option to submit one referral but request all three rehabilitation practitioners (physiatrist, physical therapist and occupational therapist) for initial and final assessments.

Participants enrolled on the yoga study to date are all female with an average age of 51years Table 1. Most participants self identified as White and Hispanic. Of those who are foreign born, time in the US ranges from 5–65 years, with varying levels of ability to speak English. The majority of the study sample has been diagnosed with rheumatoid arthritis (81.3%), the remaining participants have osteoarthritis. For this sample, they report a range of 1– 30 years with arthritis. Most (81.3%) have body mass index (BMI) measures for being either overweight or obese. Besides joint pain related to arthritis, this group of participants had previous histories of lumbar disk disease or joint replacement; or present co-morbidities of hypertension, gastric esophageal reflux, and congestive heart failure. There was a need to offer props and modifications to accommodate the various co-morbid conditions and physical limitations. Functional ability varied from those who were independent with mobility, self-care, and walked 30 minutes twice a day for exercise. To those who would need to use a chair for yoga because of a fear of not being able to get back up from the floor, or those already receiving regular physical therapy for strength and flexibility.

Facilitators for enrolling participants onto the yoga study included referral by rheumatology, previous experience with physical rehabilitation medicine, and use of complementary and alternative medicine modalities. At baseline, patients were asked to identify how many types of CAM treatments they were using based on eight categories of choices Table 1 [1,14]. On average patients in this sample reported using 4 out of 8 identified CAM modalities for arthritis.

## Preliminary Results Highlighting Partnership

For the selected physical measures the ranges and mean values for the baseline recorded measures are as follows; single leg stance 2– 30 seconds (left leg, mean = 14.7), (right leg, mean = 15.4); functional reach 10–16 inches (mean = 12.6); TUG 6–13 seconds (mean = 8.6) Table 2. Preliminary results from this study show mean baseline values for this study population had a relatively good level of functional ability before starting the intervention. Because of the small sample size, there is no attempt to determine or report statistical significance to pre and post-intervention values.

Based on the observation of preliminary outcomes, a suggestion from the clinical rehabilitation team would be to make the clinical evaluation of patients more challenging in order to evaluate significant change related to the intervention. Suggestions for modifying the measures in the future include incorporating Berg balance and quality of life measures. The timed up from floor test is viewed as a good measure of strength, flexibility, and coordination for this study. Baseline values for this measure ranged from 3–32 seconds with an average value of 10 seconds. No changes for this measure are recommended for future studies with this population and intervention.

DASH scores recorded at baseline show an interesting range of values from 2.5 to 72.5 (mean=37.7). Preliminary final assessment scores do appear to indicate positive change from baseline (data not shown). However, within this study, there is a concern that the DASH may not be as sensitive as needed. Results can be influenced by how the person is feeling that day and may not offer a true picture of change in functional ability based on the yoga intervention. Although the final measures show positive movement, an overall criticism could be that the selected measures do not have the sensitivity and specificity to identify drastic change.

When the research team met to evaluate the overall study process and preliminary results, some interesting observations emerged. In completing the DASH, a paper questionnaire, some respondents had difficulty reading the print and were confused by the spacing of the questions. The occupational therapist intervened often to assist, although many of the respondents did not readily ask for assistance. The group discussed whether this may have been a cultural response on the part of the participants or a sense of pride in not wanting to ask for assistance. However, further study would be warranted before attempting to reach any conclusions.

## Lessons Learned

When attempting to use integrative modalities with chronic diseases such as arthritis, a large self-care/management component requires a balance that may make it challenging to manage for some. Patients who are juggling doctor’s appointments, medication regimens, transportation, child-care and family needs, work schedules, and other obligations may not be able to make self-care a priority.

Based on previous experience with this population, prior to beginning the study, the therapists expressed concerns about potential no shows or difficulty meeting all required time points. Our experience to date has shown these concerns to be valid. There were several occurrences of needing to reschedule missed appointments due to inability to take time off from work, difficulty in obtaining childcare, or in needing to provide family support. Making integrative modalities more mainstreamed and affordable is important for this population, as cost and lack of knowledge may be a barrier to using integrative modalities.

In regular rehabilitation sessions, therapists observed that when patients are feeling good, they tend to overdo it and put themselves at risk for injury. This was also noticed during the yoga study. It was challenging to convince participants to use modifications that set them apart from the rest of the group even when the motivation was safety. It took considerable encouragement and condoning of the use of props until some individuals were able to progress to a less modified approach. In anticipation of this experience, yoga class was kept small. Yoga classes were offered twice a week and participants were encouraged to practice at home at least once a week. Some participants practiced at home frequently, some less often and some not at all. Due to experience with the same population in physical rehabilitation sessions, it was speculated that this could be due to lack of motivation, low priority, or wanting the instructor/therapist to make them “feel better”,however, this was not evaluated as part of this study.

This article relates the experience of collaborating with physical rehabilitation clinicians in order to select appropriate measures for a yoga intervention for a minority population. Clinical expertise was solicited in recruiting patients, selecting measures and evaluating outcomes. Combining interdisciplinary perspectives provided a synergy that augmented the quality of the study design and instruments selected for measuring changes over time. The experience of this research team of clinicians and practitioners opens the discussion for potential future interdisciplinary collaborations with rehabilitation medicine while exploring yoga as an integrative modality for minority patients with arthritis.

## Figures and Tables

**Table 1:** Selected Baseline Demographic and Clinical Measures

	Total n=16
	**Mean ± SD**
Age, years (range 40–63)	50.8 ± 8.5
Years in US (range 5–65)	25.0 ± 14.8
Years with arthritis(range 1–30)	9.0 ± 7.8
	**Percent (%)**
**ETHNICITY**	
Hispanic	81.3%
Non-Hispanic	18.8%
**RACE**	
Black/African American or Other Race	37.5%
White	62.5%
**LANGUAGE**	
English	25%
Spanish	75%
**DIAGNOSIS**	
Osteoarthritis	18.8%
Rheumatoid arthritis	81.3%

**Table 2:** Selected Measures and Assessments.

Measure	Range	Average Score
**Inventory of Complementary and Alternative Medicine (ICAMP)** Self-reported use of eight (8) possible CAM modalities for arthritis: - Health Providers and Therapists - Rubs, Liniments, Creams and Oils - Special Diets - Other Body Treatments - Vitamins and Minerals - Movement Activities - Supplements - Spiritual & Mind-Body Activities	(3–7)	4.4
**Physical Rehabilitation Assessments**		
**Single leg stance (SLS)**-(valid range=0–30 seconds) stand on one leg, place arms across chest with hands touching shoulders and do not let legs touch each other. Close eyes once in position. Evaluate with shoes off, standing about 3 feet from wall.	left (2.0–30) right (2.7–30)	left- 14.7 right- 15.4
**Functional reach** - (valid range=0–30 inches) measures the difference between arm length and maximal forward reach with the shoulder flexed to 90 degrees while maintaining fixed standing base.	(10–16)	12.6
**Timed up and go (TUG)**- (valid range=0–40 seconds) measures the time taken by an individual to stand up from a standard arm chair, walk a distance of 3 meters, turn, walk back to the chair, and sit down. ≤10 seconds=normal ≤20 seconds=good mobility, mobile without gait aid	(5.6–12.7)	8.6
**Timed Up from the Floor**(valid range=0–120 seconds) used to measure the ability to get up from the floor. Recorded in seconds then standardized using height to determine speed of task. Higher values indicate better scores.	(3.3–32.1)	10.3
**Disabilities of the Arm, Shoulder and Hand (DASH)**- a 30 item self-report questionnaire to measure physical function and symptoms in patients with musculoskeletal disorders of the upper limbs. Valid scores range from 0–100, higher scores indicate greater difficulty.	(2.5–72.5)	37.7

## List of Abbreviations

BMI: Body mass index
CAM: Complementary and alternative medicine
CHC: Community health center
DASH: Disabilities of the Arm Shoulder, and Hand
ICAMP: Inventory of Complementary and Alternative Medicine Practices
NIH: National Institutes of Health
NIAMS: National Institute of Arthritis and Musculoskeletal and Skin Diseases
OA: Osteoarthritis
RA: Rheumatoid arthritis
RMD: Rehabilitation Medicine Department
SLS: Single leg stance
TUG: Timed “*Up and Go*” *test*
